# Surface Layer Protein A Expressed in *Clostridioides difficile* DJNS06-36 Possesses an Encephalitogenic Mimotope of Myelin Basic Protein

**DOI:** 10.3390/microorganisms9010034

**Published:** 2020-12-24

**Authors:** John E. Mindur, Sudhir K. Yadav, Naoko Ito, Mitsutoshi Senoh, Haru Kato, Suhayl Dhib-Jalbut, Kouichi Ito

**Affiliations:** 1 Department of Neurology, Rutgers-Robert Wood Johnson Medical School, Piscataway, NJ 08854, USA; jmindur@g.harvard.edu (J.E.M.); yadavsk@rwjms.rutgers.edu (S.K.Y.); itona@rwjms.rutgers.edu (N.I.); jalbutsu@rwjms.rutgers.edu (S.D.-J.); 2 Center for Systems Biology, Massachusetts General Hospital and Harvard Medical School, Boston, MA 02114, USA; 3 Department of Bacteriology II, National Institute of Infectious Diseases, Tokyo 208-001, Japan; senoh@nih.go.jp (M.S.); cato@nih.go.jp (H.K.)

**Keywords:** *Clostridioides difficile*, surface layer protein A, multiple sclerosis, myelin basic protein, mimotope

## Abstract

Multiple sclerosis (MS) is an inflammatory demyelinating disease of the central nervous system (CNS). Recent studies suggest that migration of Th1 and Th17 cells specific for enteric bacteria from the gut to the CNS may lead to the initiation and/or exacerbation of autoimmune diseases including MS. Human leukocyte antigen (HLA)-DR15 is an MHC class II (MHCII) haplotype highly associated with the development of MS that contains the two HLA-DRB* genes, DRB1*1501 (DR2b) and DRB5*0101 (DR2a). To identify enteric bacteria which harbor antigenic epitopes that activate myelin-specific T cells and drive CNS inflammation, we screened for enteric bacteria which express cross-reactive epitopes (‘mimotopes’) of an immunodominant myelin basic protein 89–98 (MBP_89-98_) epitope. Based on known MHCII HLA-DR2a amino acid binding motifs and cultivation with splenic T cells isolated from MBP-T cell receptor (TCR)/DR2a transgenic (Tg) mice, we discovered that a certain variant of surface layer protein A (SLPA), which is expressed by a subtype of *Clostridioides difficile*, contains an amino acid sequence that activates MBP_89-98_-reactive T cells. Furthermore, activation of MBP-specific T cells by SLPA upon active immunization induced experimental autoimmune encephalomyelitis (EAE) in MBP-TCR/DR2a Tg mice. This study suggests that a unique strain of *C. difficile* possesses an encephalitogenic mimotope of MBP that activates autoreactive, myelin-specific T cells.

## 1. Introduction

Multiple sclerosis (MS) is an immune-mediated inflammatory and demyelinating disease of the central nervous system (CNS) [1]. The differentiation and migration of myelin-specific Th1 and Th17 cells from the gut tissues into the CNS has been suggested as an initial event in MS pathogenesis. Although myelin-specific T cells are present in healthy individuals, regulatory immune cells suppress their differentiation into encephalitogenic cells. However, infections by microorganisms may sometimes break immune tolerance and potentially favor myelin-specific T cell escape from immune tolerance mechanisms. Molecular mimicry is one hypothesis that has been proposed as a mechanism to explain how autoreactive T cells are able to differentiate into pathogenic cells that drive autoimmune disease development [2]. Typically, foreign and self-antigens (or autoantigens) are processed and presented by antigen-presenting cells (APCs) as peptide fragments and CD4^+^ T cells recognize these processed peptide sequences, which are comprised of approximately 10–16 amino acids, by binding to MHC class II (MHCII) molecules via their T cell receptor (TCR). Peptide epitopes comprised of similar amino acids bearing a structural resemblance to autoantigen epitopes, or ‘mimotopes’, can also bind to specific pockets within MHCII upon antigen processing, and these mimotope:MHCII complexes may activate autoreactive T cells [3,4,5,6].

Although the etiology of MS is still unknown, it has been suggested that genetic and environmental elements are mutually involved in the initiation and progression of MS [1]. Among MS-associated genetic elements, the MHCII HLA-DR gene is the most strongly associated element [7]. HLA-DR15, containing the DRB1*1501 (DR2b) and DRB5*0101 (DR2a) genes, is highly associated with MS, suggesting that DR2a- or DR2b-restricted T cells may be involved in MS pathogenesis. Among environmental factors, gut dysbiosis (i.e., an alteration among gut microbial species) has been reported as a major MS-associated factor [8,9,10,11]. Studies on gut dysbiosis in animal models of MS suggest the relative expansion of pathogenic microbes reduces the abundance of bacteria that promote Foxp3^+^ Treg development (e.g., *Clostridium* and *Prevotella* species), therefore increasing the risk of MS [12,13]. Additionally, gut dysbiosis increases intestinal permeability and subsequently induces systemic circulation of pathogen-associated molecular patterns (PAMPs) produced by enteric bacteria, which increases the degree of systemic and CNS inflammation [14].

Recent studies also suggest that pathogenic Th17 cells can migrate into other organs from the gut [13]. Importantly, Th17 cells specific for microbial components can migrate into peripheral lymphoid organs and initiate autoimmunity [15]. Although T cells undergo tolerance to gut bacteria, the expansion of pathogenic intestinal bacteria may break this immune tolerance and induce the development of T cells specific for enteric bacterial antigens. In turn, gut bacteria-specific T cells may migrate into the blood circulation. Indeed, gut microbiota-specific T cells are detected in the blood of IBD patients [16]. Even in healthy individuals, gut microbiota-reactive T cells can be detected in the blood [17]. Therefore, if T cells specific for enteric bacteria happen to encounter APCs presenting CNS autoantigens and cross-react to these autoantigens, their activation and migration into peripheral lymphoid organs and the CNS are high-risk cellular events for CNS autoimmunity. MBP_89-98_ is an immunodominant T cell epitope of MBP in HLA-DR15^+^ individuals [18]. We previously showed that DR2a-restricted MBP_89-98_-specific Th17 cells are highly encephalitogenic in DR2a Tg mice [19,20]. In this study, we investigated whether enteric bacteria express mimotopes of MBP_89-98_. We searched for putative mimotope candidate peptide sequences through the NCBI Microbial Protein Database based on known HLA-DR2a antigenic binding motifs, and upon screening the 28 putative candidates, found that surface layer protein A (SLPA) expressed in a subset of *Clostridioides difficile* possesses encephalitogenic mimotope of MBP_89-98_.

## 2. Materials and Methods

### 2.1. Determination of Candidate Bacterial Mimicry Peptide Epitopes for Screening

Proteins encoding potential MBP_89-98_ mimotope sequences were determined by searching through Microbial Protein BLAST by convention of the Protein BLAST program (BLASTP) available through NIH NCBI. The explored genomes were limited to microbial species that can colonize humans and mice. Therefore, the search set was narrowed down to genera consisting of Bacteroides (taxid:816), Clostridium (taxid:1485), Fusobacterium (taxid:848), Eubacterium (taxid:1730), Ruminococcus (taxid:1263), Peptococcus (taxid:2740), Peptostreptococcus (taxid:1257), Bifidobacterium (taxid:1678), Escherichia (taxid:561) and Lactobacillus (taxid:1578) [21]. For the listed genera, a total of 41,724,149 sequences were screened. The amino acid queries for candidate mimotopes were designed to share sequence homology with the immunodominant MBP_89-98_ epitope, particularly for residues 89-98 (FFKNIVTPRT), since these amino acid residues structurally fit into the MHCII HLA-DR2a binding pocket [22] and can establish contact with the 3A6 T cell receptor (TCR) of myelin-reactive MBP_89-98_-specific T cells [19,23]. The candidate mimotope sequences were further selected based on their predicted MHCII binding capacity for HLA-DR2a (HLA-DRB5*01:01) using the IEDB Analysis Resource Consensus Tool (http://tools.iedb.org/mhcii/) [24,25]. Candidate mimotope peptides (Peptide 2.0 Inc., Chantilly, VA, USA) were finally screened by T cell proliferation assays using MBP_89-98_-specific T cells.

### 2.2. Isolation and Characterization of Clostridioides difficile

*C. difficile* strain DJNS06-36 was identified as PCR ribotype (RT) gc0636. It was isolated from the stool of an 83-year-old woman on 11/24/2005 in Gifu, Japan. She presented to her general practitioner with diarrhea and abdominal cramping and was admitted to hospital. She had taken tosufloxacin for her upper respiratory infection as outpatient treatment. On admission, laboratory testing for toxin in feces was positive, and she was given the diagnosis of *Clostridioides difficile* infection (CDI) [26].

*C. difficile* strain JND10-141 was identified as a unique RTcc10141 strain. It was isolated from the stool of an 80-year-old woman who was hospitalized for her meningioma on 3/29/2010 in Chiba, Japan. She suffered from CDI and recurrence during hospitalization. JND10-141 was isolated from the second episode (recurrence).

### 2.3. Generation of Recombinant E. coli Expressing MBP, SLPA, or LacZ

MBP, SLPA, and LacZ cDNA were introduced into the Champion™ pET Directional TOPO^®^ Expression Kit (Invitrogen, Carlsbad, CA, USA). BL21 Star™ (DE3) *E. coli* was transformed with the recombinant vectors, grown, and then their expression was induced by the addition of 1 mM IPTG into growth media according to manufacturer instructions. The recombinant His(6X)-tagged proteins were subsequently purified utilizing the ProBond™ Purification System (Invitrogen). The eluted protein was concentrated by PES, 3K MWCO (ThermoFisher Scientific, Waltham, MA, USA). Final concentration of protein was measured using the Pierce™ BCA Protein Assay Kit (ThermoFisher Scientific).

### 2.4. Cell Culture, T Cell Proliferation and Flow Cytometry

To screen for mimotope peptides of MBP_89-98_, splenocytes isolated from MBP-TCR/DR2a, Tg mice were cultured with candidate peptides at 10 μg/mL and cell proliferation was measured by ^3^H thymidine uptake using a TriLux Liquid Scintillation Counter (Wallac). To measure IL-17A production in response to *C. difficile* and recombinant *E. coli* lysates, MBP_89-98_-specific Th17 cells were generated by cultivation of splenocytes isolated from MBP-TCR/DR2a Tg mice with IL-6, TGF-beta, and IL-23 as previously described [27]. Dendritic cells (DCs) isolated from DR2a Tg mice were stimulated with Pam3CSK4 at 10 ng/mL for 2 days. MBP_89-98_-specific Th17 cells were cultured with the DR2a^+^ DCs and *C. difficile* (30 μg/mL) or recombinant *E. coli* (60 μg/mL) lysates. *C. difficile* strains DJNS06-36 and JND10-141 recovered from patients who suffered from *C. difficile* infection were used as described above. Cell lysates were prepared by sonication of bacterial suspensions in PBS. To measure the response to SLPA, memory T cells were isolated from MBP-TCR/DR2a Tg mice with memory T cell isolation microbeads (Miltenyi Biotec, Auburn, CA, USA) and then cultured with DR2a^+^ DCs and SLPA at 70 μg/mL in the presence or absence of anti-HLA-DR monoclonal antibody (mAb) (10 μg/mL). Production of IL-17A was measured by ELISA (BioLegend, San Diego, CA, USA). To examine the expression of IL-17A, GM-CSF, and IFN-γ in the brain and spinal cord, CNS cell infiltrates were purified with a 30/70% percoll-density gradient after digestion of the homogenized brain and spinal cord with Neural Tissue Dissociation kit (Miltenyi Biotec, Auburn, CA, USA). The isolated CNS cells were cultured with MBP_89-98_ at 10 μg/mL for 5 h in the presence of brefeldin A (10 μg/mL) and then stained with anti-CD4, -CD3, -IL-17A, -GM-CSF, and -IFN-γ mAbs. Anti-CD4, -IFN-γ, -IL-17A, and -GM-CSF mAbs were all purchased from eBioscience and mAbs against human 3A6 TCR Vβ5.1 were purchased from Beckman Coulter. Flow cytometry analysis was performed on a Gallios flow cytometer and analyzed by Kaluza Software (Beckman Coulter, Brea, CA, USA).

### 2.5. Mice and EAE Induction of Experimental Autoimmune Encephalomyelitis (EAE)

MBP-TCR/DR2a Tg mice were immunized s.c. with 200 μg of SLPA in Complete Freund’s Adjuvant (CFA) (Difco Laboratories, Detroit, MI, USA) on day 0 and 200 ng of pertussis toxin (List Biologicals, St. Louis, MO, USA) was administered i.p. on days 0 and 2 for the induction of EAE. Animals were weighed and monitored daily; clinical signs were assessed according to the following scale: 0: no disease; 1: limp tail; 2: mild hindlimb paresis; 2.5: severe hindlimb paresis; 3: single hindlimb paralysis; 3.5: complete hindlimb paralysis; 4: hindlimb paralysis and forelimb paresis; and 5: no mobility/moribund. All experiments were carried out in compliance with the Guide for the Care and Use of Laboratory Animal Resources and approved by the Rutgers-Robert Wood Johnson Medical School Institutional Animal Care and Use Committee.

## 3. Results

### 3.1. Screening of Gut Microbes for MBP_89-98_ Mimotope Peptides

**Figure 1:**
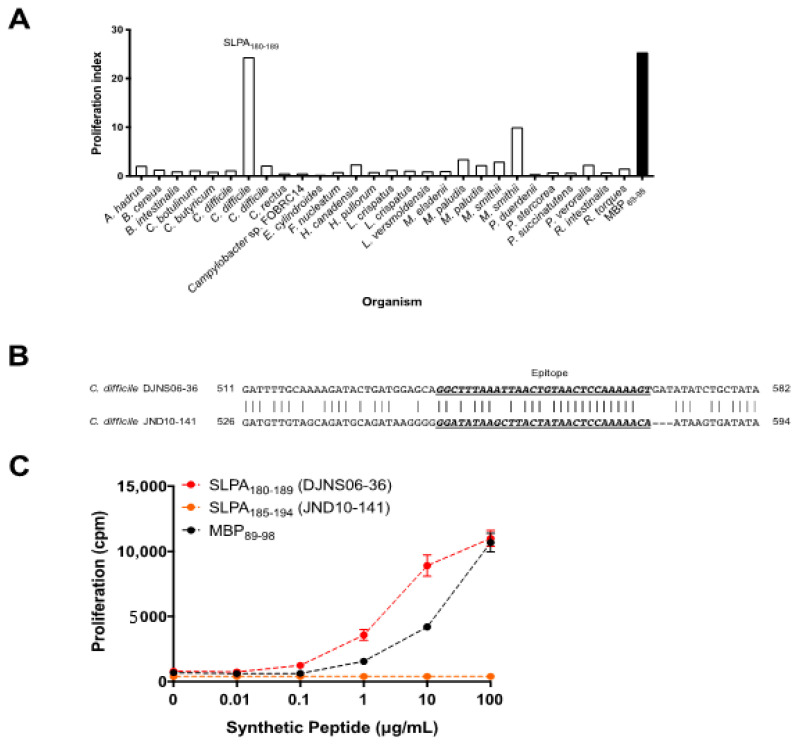
SLPA expressed by *C. difficile* DJNS06-36 possesses an MBP mimotope for DR2a-restricted MBP_89-98_-specific T cells. (**A**) Proliferation of DR2a-restricted MBP-specific Tg T cells in response to synthetic enteric bacteria peptide sequences. Mean of two experiments is presented. (**B**) Difference in DNA sequence for the MBP mimotope between *C. difficile* DJNS06-36 and *C. difficile* JND10-141. (**C**) Synthetic *C. difficile* DJNS06-36 SLPA_180-189_ peptide mimotope of MBP_89-98_ for proliferation of DR2a-restricted MBP-specific T cells in comparison to MBP_89-98_ and synthetic *C. difficile* JND10-141 SLPA_185-194_ peptide. Data from one representative experiment of three independent experiments is presented.

MBP is a CNS autoantigen in MS and MBP_89-98_ is one of the encephalitogenic epitopes of MBP that can be recognized by myelin-reactive T cells in the context of MHCII HLA-DR2a antigen presentation. HLA-DR2a is associated with MS and HLA-DR2a-restricted MBP_89-98_ T cells can be isolated from MS patients [28]. Therefore, we investigated whether microbial proteins derived from intestinal microbes can act as mimotopes to activate MBP_89-98_-specific T cells. We first screened for potential mimotopes of MBP_89-98_ amongst enteric microbial proteins through the NCBI Microbial Protein Database based on the amino acids of MBP_89-98_ that act as MHCII HLA-DR2a antigenic binding motifs [29]. We subsequently found 28 microbial candidates that expressed potential MBP_89-98_ mimotopes (Table 1). Next, we examined which of the identified enteric bacterial protein(s) could stimulate MBP_89-98_ reactive T cells. We utilized splenocytes isolated from humanized transgenic mice that express an MBP_89-98_-specific human TCR and HLA-DR2a, and are deficient in expression of the endogenous mouse MHCII (I-E and I-A) [19,20]. We generated synthetic decapeptides for each candidate mimotope and screened for cellular proliferation upon culturing the MBP-TCR/DR2a Tg splenocytes with candidate mimotope peptides. T cell proliferation as assessed by thymidine uptake revealed that synthetic *C. difficile* DJNS06-36 surface layer protein A (SLPA) peptide strongly induced the proliferation of MBP_89-98_-specific T cells (Figure 1A). 

### 3.2. Diversity amongst the SLPA Mimotopes in C. difficile Strains

Notably, SLPA is an adhesion protein expressed by *C. difficile* that mediates binding and adherence to extracellular matrix proteins and gastrointestinal tissues [30,31,32]. It is composed of a conserved high-molecular-weight subunit and low-molecular-weight subunit, the latter of which is diverse among *C. difficile* strains [33,34,35,36]. As shown in Figure 1B, the DNA sequence of SLPA is different between *C. difficile* DJNS06-36 and *C. difficile* JND10-141. As a result, the amino acid sequence of the SLPA mimicry epitope is different (Table 2). While the *C. difficile* DJNS06-36 genome encodes for the SLPA_180-189_ peptide (GFKLTVTPKS) according to NCBI Microbial Protein Database, the *C. difficile* JND10-141 genome encodes for SLPA_185-194_ peptide (GYKLTITPKT). To test whether the proliferative response is restricted to synthetic *C. difficile* DJNS06-36 SLPA_180-189_ peptide, we evaluated cellular proliferation in response to both of the SLPA peptide epitopes. Indeed, MBP_89-98_-specific T cells proliferated in response to synthetic *C. difficile* DJNS06-36 SLPA_180-189_ peptide; however, synthetic *C. difficile* JND10-141 SLPA_185-194_ did not trigger a proliferative response (Figure 1C). This data indicates that the HLA-DR2a-restricted myelin-reactive T cell response is specific to SLPA expressed in a certain strain of *C. difficile*.

### 3.3. The SLPA Mimotope Can Be Processed and Presented to MBP_89-98_-Specific T Cells

Although the potent SLPA_180-189_ mimotope peptide could effectively trigger MBP_89-98_-specific T cell proliferation, it remained unclear whether the entire mimotope could be efficiently processed and presented by MHCII HLA-DR2a on antigen-presenting cells (APCs) following uptake of bacteria containing the SLPA_180-189_ epitope. Thus, we next assessed whether co-culture of bacteria encoding the SLPA_180-189_ epitope with HLA-DR2a^+^ CD11c^+^ dendritic cells (DCs) could trigger the activation of MBP_89-98_-specific T cells and their subsequent production of pro-inflammatory mediators.

**Figure 2:**
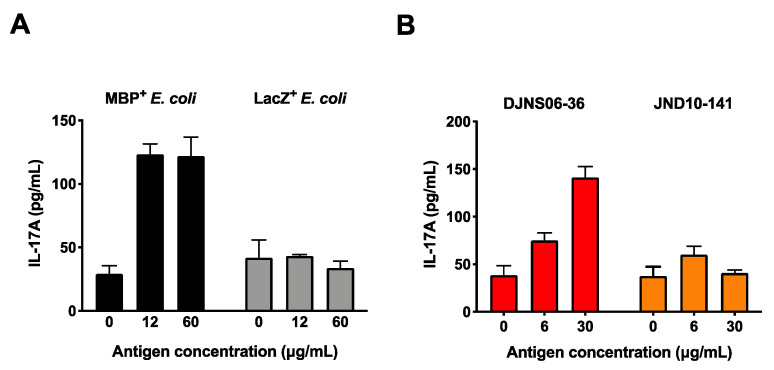
Production of IL-17A in MBP-specific Th17 cells in response to *C. difficile* DJNS06-36. MBP-specific Th17 cells were cultured with DR2a^+^ DCs pulsed with cell extract of either (**A**) MBP^+^ *E. coli* and LacZ^+^ *E.coli* or (**B**) *C. difficile* DJNS06-36 and *C. difficile* JND10-141, and the production of IL-17A was examined. Data from one representative experiment of two independent experiments are presented.

First, we generated *Escherichia coli* expressing the MBP_89-98_ epitope (positive control) or an unrelated LacZ sequence (negative control) by transformation of *E. coli* with MBP or LacZ genes, respectively. MBP_89-98_-specific Th17 cells were generated by cultivation of splenocytes isolated from MBP-TCR/DR2a Tg mice with IL-6, TGF-beta, and IL-23. To confirm that IL-17A can be produced by myelin-specific T cell upon cultivation of bacteria expressing MBP_89-98_, MBP_89-98_-specific Th17 cells were cultured with HLA-DR2a^+^ CD11c^+^ DCs pre-incubated with the MBP^+^ or LacZ^+^ *E. coli* lysates. As shown in Figure 2A, production of IL-17A was detected when MBP_89-98_-specific T cells were co-cultured with DCs exposed to MBP^+^ *E. coli* but not to LacZ^+^ *E. coli*, indicating that IL-17A can be produced in response to the MBP_89-98_ epitope following antigen processing of MBP in this *in vitro* system. Next, we examined whether *C. difficile* DJNS06-36 SLPA_180-189_ can be processed and presented to MBP_89-98_ specific Th17 cells. Notably, cultivation of MBP_89-98_-specific Th17 cells with HLA-DR2a^+^ CD11c^+^ DCs exposed to *C. difficile* DJNS06-36 could induce the production of IL-17A (Figure 2B). On the other hand, MBP_89-98_-specific T cells could not produce IL-17A when co-cultured with HLA-DR2a^+^ CD11c^+^ DCs exposed to *C. difficile* JND10-141, likely because this strain lacks the potent MBP_89-98_ mimotope.

**Figure 3:**
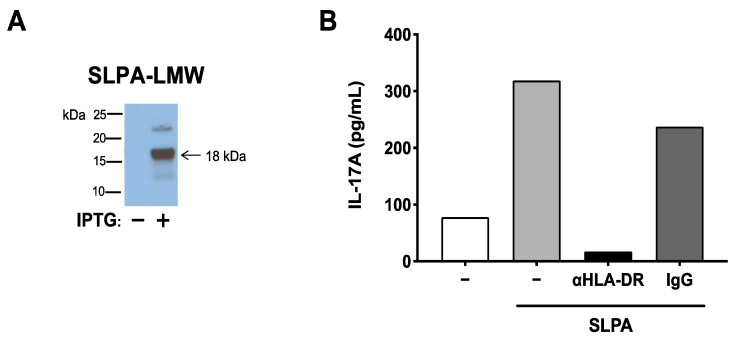
SLPA produced by *C. difficile* DJNS06-36 can be processed and presented to DR2a-restricted MBP_89-98_-specific T cells. (**A**) Purification of LMW-SLPA from BL21 Star™ (DE3) *E. coli* that expresses LMW-SLPA DNA isolated from *C. difficile* DJNS06-36. His(6X)-tagged LMW-SLPA protein was induced by IPTG. (**B**) Memory T cells isolated from MBP-TCR/DR2a Tg mice were cultured with the purified LMW-SLPA in the presence of anti-HLA-DR mAb or isotype control IgG. IL-17A production in response to LMW-SLPA in the context of DR2a was examined. Data from one representative experiment of two independent experiments is presented.

SLPA consists of low- and high-molecular-weight S-layer protein A (LMW-SLPA and HMW-SLPA). Since the MBP_89-98_ mimotope is found in the genetic sequence that encodes for LMW-SLPA, *E. coli* was transformed with LMW-SLPA DNA isolated from *C. difficile* DJNS06-36, and LMW-SLPA was subsequently purified from the LMW-SLPA^+^ *E. coli* (Figure 3A). To confirm that LMW-SLPA derived from *C. difficile* DJNS06-36 can be processed and presented to MBP_89-98_-specific T cells by HLA-DR2a, the purified LMW-SLPA was cultured with HLA-DR2a^+^ CD11c^+^ DCs and MBP_89-98_-specific T cells in the presence of HLA-DR monoclonal antibody (mAb) or isotype control IgG. As shown in Figure 3B, IL-17A production in response to LMW-SLPA was blocked by anti-HLA-DR mAb but not by isotype control IgG, suggesting that LMW-SLPA can be processed and presented to DR2A-restricted MBP_89-98_-specific T cells.

### 3.4. Induction of EAE in MBP-TCR/DR2a Tg Mice upon Immunization with Purified SLPA

**Figure 4:**
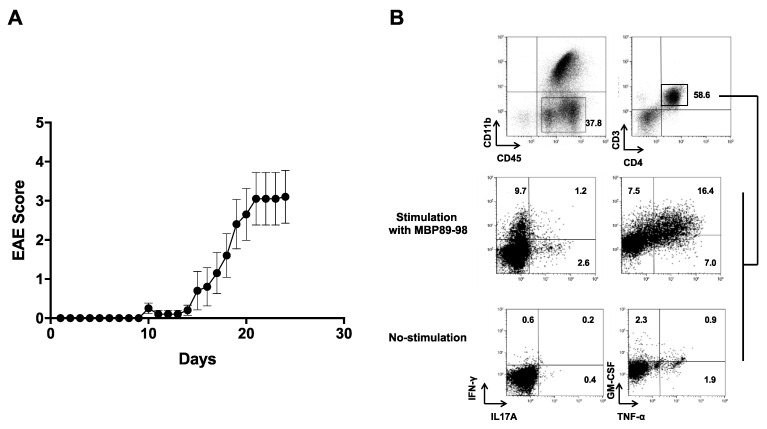
Induction of EAE in MBP-TCR/DR2a Tg mice upon immunization with *C. difficile* DJNS06-36 SLPA protein. (**A**) MBP-TCR/DR2a Tg mice (*n* = 10) were immunized with LMW-SLPA/CFA and development of EAE was examined. The combined data from two independent experiments is presented. (**B**) Development of Th1 and Th17 cells in EAE mice. Spinal cords isolated from EAE mice were stained with CD11b, CD45, CD4, CD3, IL-17A, and IFN-γ mAbs. CD45^+^ CD11b^−^ CD3^+^ CD4^+^ T cells were gated and examined for development of Th1 (IFN-γ^+^), Th17 (IL-17A^+^), and ThGM (GM-CSF^+^) cells.

Since MBP_89-98_ is one of the encephalitogenic epitopes in MBP, we next assessed whether *C. difficile* DJNS06-36 LMW-SLPA is an encephalitogenic protein in MBP-TCR/DR2a Tg mice upon active immunization. We thus induced EAE by immunizing MBP-TCR/DR2a Tg mice with the purified LMW-SLPA and monitored the mice for clinical signs of EAE. As expected, we observed the induction of EAE upon immunization with LMW-SLPA/CFA, including the infiltration of MBP_89-98_-specific Th1 (IFN-γ^+^), Th17 (IL-17A^+^), and ThGM (GM-CSF^+^) cells in the CNS (Figure 4). Therefore, *C. difficile* DJNS06-36 SLPA can trigger EAE in mice harboring myelin-reactive, MBP_89-98_-specific T cells. This data suggests that infection with *C. difficile* DJNS06-36 may increase the risk of CNS autoimmunity in HLA-DR2a^+^ individuals.

## 4. Discussion

Microbial infections have long been suggested to be involved in triggering MS [37]. Molecular mimicry is one potential mechanism that explains how infectious agents and/or microbes might induce immune-mediated diseases such as MS [38,39]. This hypothesis proposes that self-reactive T and/or B cells that drive immune-mediated diseases initially encounter and become activated by bacteria or viruses possessing shared antigenic determinants, or mimotopes, with host self-antigens. MBP_89-98_ is one of the encephalitogenic autoantigens and MBP-specific T cells are present in healthy individuals. Although myelin-reactive T cells undergo central and peripheral tolerance [40], an environmental stimulus that can induce the development of myelin-specific encephalitogenic Th1 or Th17 cells could be a risk factor for MS. An early MS study showed that mimotopes of MBP are expressed in certain types of viruses, such as hepatitis B virus, which encodes an encephalitogenic epitope within the hepatitis virus B polymerase [2]. Epstein-Bar virus (EBV) is also a well-known MS-associated virus and MBP-specific T cells cross-react to EBV peptides [41,42]. Another study showed that MBP-specific antibodies purified from MS patients cross-react to EBV latent membrane protein 1 [43]. Therefore, EBV-derived MBP mimotopes may be involved in disease initiation and/or progression of MS.

Recent studies suggest that gut dysbiosis reduces intestinal microbiota species involved in the development of Foxp3^+^ Tregs [44]. This dysbiosis-mediated reduction in regulatory cells could increase the risk of developing MS. In addition, DCs sample luminal bacteria antigens and induce the development of Th1 and Th17 cells specific for enteric bacteria antigens [45]. Indeed, intestinal microbiota-specific memory CD4^+^ T cells are abundant in the peripheral blood [17]. Importantly, blood circulation of microbial protein-specific T cells increases upon induction of gut dysbiosis-mediated intestinal inflammation [16]. If colonization of gut bacteria expressing an MBP mimotope is elevated, an increase in blood circulating MBP mimotope-reactive T cells and MBP mimotope^+^ DCs may increase the risk of MS. Therefore, we investigated whether MBP mimotopes are expressed in enteric bacteria. We showed here that SLPA_180-189_ found in *C. difficile* DJNS06-36 possesses an MBP_89-98_ mimotope. Since MBP-TCR/DR2a Tg mice express a single TCR specific for MBP_89-98_/DR2a, future studies are necessary to examine the frequency of MBP_89-98_-reactive T cells that can respond to DJNS06-36 SLPA_180-189_:DR2a complexes in HLA-DR2a^+^ individuals.

*C. difficile* is a well-known anaerobic bacterium that can be pathogenic in the gut, as it can cause nosocomial antibiotic-associated diarrhea and colitis in humans [46]. However, *C. difficile* does not always cause diarrhea and colitis. Approximately 4.0–7.6% of healthy adults actually carry *C. difficile* bacteria in their gut [47,48]. Since non-toxigenic strains also express SLPA, non-toxigenic *C. difficile* expressing an MBP_89-98_ mimotope may increase the risk of MS. SLPA is the most abundant surface protein on *C. difficile* that interacts with intestinal epithelial cells. Among *C. difficile* subtypes, the protein sequence of SLPA is heterogenous [49,50]. SLPA consists of low- and high-molecular-weight S-layer protein A (LMW-SLPA and HMW-SLPA, respectively) and LMW-SLPA has a heterogenous sequence [26,51]. *C. difficile* DJNS06-36 expresses an MBP_89-98_ mimotope, whereas *C. difficile* JND10-141 does not express the mimotope. This difference is due to heterogenicity in LMW-SLPA as shown in Figure 1B and Table 2. We further produced LMW-SLPA from *C. difficile* DJNS06-36 and found that LMW-SLPA expressed in *C. difficile* DJNS06-36 contains an MBP_89-98_ mimotope upon co-culture with myelin-reactive, MBP-specific T cells. Since *C. difficile* was reportedly able to colonize antibiotic treated C57BL/6 mice [52], we inoculated *C. difficile* DJNS06-36 into MBP-TCR/DR2a Tg mice after antibiotic treatment to evaluate their frequency of EAE development. However, these animals failed to develop EAE (data not shown). Since weight loss and intestinal inflammation were minimal in the inoculated MBP-TCR/DR2a Tg mice, the colonization of MBP-TCR/DR2a Tg mice with *C. difficile* DJNS06-36 may be insufficient.

Importantly, SLPAs are involved in activation of innate cells through binding to Toll-like receptor (TLR) [53,54], and TLR stimulation induces the maturation of DCs and subsequently induces the development of Th1 and Th17 cells [35]. Therefore, the development of SLPA-specific Th17 cells may play a role in the clearance of *C. difficile*. The SLPA-activated DCs may also differentiate into pathogenic DCs that present the MBP_89-98_ mimotope, and their migration into systemic circulation may contribute to the development of CNS autoimmunity. However, it is still unknown whether infection with *C. difficile* DJNS06-36 increases the risk of MS development in humans. Since *C. difficile* DJNS06-36 RTgc0636 has been rarely detected in Japan [26,55,56], we further investigated the NCBI Microbial Protein Database with the SLPA mimotope and found that the SLPA mimotope is expressed in three other *C. difficile* strains, JND 08-232 (RTaz08232), strain 630 (RT012), and strain RIGLD-284 (RT not reported), isolated in Japan, Switzerland, and Iran, respectively [57,58]. Future studies are necessary to explore the association between infections caused by these *C. difficile* strains and CNS autoimmunity in HLA-DR15^+^ individuals.

## Figures and Tables

**Table 1:** Candidate peptide mimotopes of gut microbes.

Organism	Name of Gene Encoding Candidate Mimotope	Peptide Sequence										NCBI Protein Accession
*Anaerostipes hadrus*	Riboflavin biosynthesis protein RibF	D	F	K	L	I	V	I	P	K	L	ZP_19295054.1
*Bacillus cereus*	Ger(X)C family germination protein	R	Y	K	L	T	I	T	P	K	E	ZP_17590229
*Bacteroides intestinalis*	ATPase/histidine kinase/DNA gyrase B/HSP90 domain	A	K	K	L	M	L	S	K	R	K	EDV06450.1
*Clostridium botulinum*	Putative histidine kinase	F	Y	K	L	V	L	S	K	R	N	YP_004385843.1
*Clostridium butyricum*	Integral membrane protein domain protein	A	F	K	L	L	K	T	K	K	G	ZP_02949557
*Clostridium difficile*	Biotin/lipoate A/B protein ligase family protein	L	F	K	L	I	K	T	K	T	P	ZP_17070733
*Clostridium difficile*	Surface layer protein A	G	F	K	L	T	V	T	P	K	S	BAF02835
*Clostridium difficile*	Cell wall-binding repeat 2 family protein	G	Y	K	L	T	I	T	P	K	T	WP_021360901
*Campylobacter rectus*	Isoleucyl-tRNA synthetase	K	F	K	L	V	L	S	T	R	H	ZP_03610955.1
*Campylobacter* sp. FOBRC14	tRNA pseudouridine(38-40) synthase	F	G	K	L	V	L	S	S	R	T	ZP_10843097.1
*Eubacterium cylindroides*	Glycosyltransferases involved in cell wall biogenesis	F	Y	K	L	I	K	T	K	K	A	CBK88797.1
*Fusobacterium nucleatum*	Integrase/recombinase	F	F	K	L	I	Q	T	K	S	G	ZP_06750855.1
*Helicobacter canadensis*	Glutathionylspermidine synthase	F	F	K	N	M	V	I	L	K	F	ZP_04870684.1
*Helicobacter pullorum*	Glutathionylspermidine synthase	W	F	K	L	I	P	W	E	S	I	WP_065826614
*Lactobacillus crispatus*	Transporter, major facilitator family	V	F	K	N	I	K	T	R	T	K	ZP_06627878.1
*Lactobacillus crispatus*	Transporter, major facilitator family	Y	Y	K	P	V	T	P	K	K	T	ZP_06627878.1
*Lactobacillus versmoldensis*	HNH endonuclease	D	Y	K	L	I	K	T	K	K	G	WP_040521112
*Megasphaera elsdenii*	CRISPR-associated protein	A	F	K	L	M	K	T	K	K	P	CCC73991
*Methanobacterium paludis*	MtaA/CmuA family methyltransferase	Q	F	K	S	I	V	K	P	R	L	WP_013825607
*Methanobacterium paludis*	Thioesterase	M	F	K	T	V	V	T	P	R	F	WP_013825042
*Methanobrevibacter smithii*	DEXX-box ATPase	M	F	K	R	V	V	T	P	L	N	WP_004032273
*Methanobrevibacter smithii*	Thioesterase	M	F	R	T	I	V	T	P	K	F	WP_011953703
*Peptoniphilus duerdenii*	Transcriptional regulator	N	F	K	L	V	K	T	K	K	A	WP_008901584
*Prevotella stercorea*	Chain length determinant protein	V	F	K	L	L	K	T	K	K	K	CDE30894
*Phascolarctobacterium succinatutens*	DNA topoisomerase I	A	K	K	T	I	V	T	K	K	T	ZP_08075451.1
*Prevotella veroralis*	Glycine cleavage system T protein	G	Y	R	L	I	S	T	P	K	S	ZP_05856218
*Roseburia intestinalis*	Lysine--tRNA ligase	F	K	K	N	I	V	T	K	T	Y	ZP_04745032.1
*Ruminococcus torques*	Guanine deaminase	K	Y	K	N	T	L	P	I	L	T	CUN31532
*Homo sapiens*	Myelin basic protein/Golli-Myelin basic protein	F	F	K	N	I	V	T	P	R	T	NP_001020272
*Mus musculus*	Myelin basic protein/Golli-Myelin basic protein	F	F	K	N	I	V	T	P	R	T	NP_034907

**Table 2:** Peptide sequences of SLPA and MBP epitopes.

Organisms	Peptide					Peptide Sequence						NCBI Protein Accession
*C. difficile* DJNS 06-36	SLPA_180-189_	G	F	K	L	T	V	T	P	K	S	BAF02835.1
*C. difficile* JND 10-141	SLPA_185-194_	G	Y	K	L	T	I	T	P	K	T	BAM66401.1
*Homo sapiens*	MBP_89-98_	F	F	K	N	I	V	T	P	R	T	CAG46717.1
*Mus musculus*	MBP_87-96_	F	F	K	N	I	V	T	P	R	T	NP_034907

## Data Availability

Data is contained within the article and can be made further available upon request from the corresponding author.
